# Cybervictimization and Online Sexual Harassment: Prevalence, Association, and Predictors

**DOI:** 10.3390/ijerph21121555

**Published:** 2024-11-25

**Authors:** Angela Franceschi, Lisa De Luca, Annalaura Nocentini, Ersilia Menesini

**Affiliations:** Department of Education, Languages, Intercultures, Literatures and Psychology, University of Florence, Via di San Salvi, 12, Complesso di San Salvi Padiglione 26, 50135 Florence, Italy; angela.franceschi@unifi.it (A.F.); annalaura.nocentini@unifi.it (A.N.); ersilia.menesini@unifi.it (E.M.)

**Keywords:** cybervictimization, online sexual harassment, school climate, adolescents

## Abstract

Background: The daily and massive use of the Internet and social media by adolescents has led to increased interest and attention to prevalence rates, risk factors, and potential consequences of different forms of online victimization. This study aims to examine the possible associations between cybervictimization and online sexual harassment among 697 Italian adolescents (M_age_ = 15.17; SD = 0.68; 42.3% female), understanding the contribution of individual and school risk factors. Methods: A short longitudinal design was used to test a path model where emotional/behavioral problems and school climate predicted cybervictimization and online sexual harassment, controlling for their co-occurrence. Results: The results show similar prevalence among the two phenomena with a consistent reciprocal association (*ρ*s = 0.426**). Regarding predictors, cybervictimization at Wave 5 is predicted by the problematic peer relationships with peers (β = 0.164*, SE = 0.068) and lack of school cohesion (β = −0.189*, SE = 0.086) assessed at Wave 4. In contrast, online sexual harassment at Wave 5 is predicted by the presence of emotional symptoms (β = 0.248***, SE = 0.077) and the absence of social norms (β = −0.254**, SE = 0.085) measured at Wave 4. Conclusion: Online sexual harassment and cybervictimization are related phenomena with a co-occurrence of around 22%; being a victim of cybervictimization is positively associated with being a victim of OSH-P. However, risk factors are different: cybervictimization is more easily explained by social and contextual factors, while online sexual harassment is explained by individual factors.

## 1. Introduction

The daily and massive use of the Internet and social media by youth has led to an ever-increasing interest and attention to the rates, risk factors, and potential consequences of online victimization [1,2,3,4,5,6,7,8]. Some phenomena, such as cyberbullying, have long been studied and researched, while others, such as online sexual harassment, are still less known [9]. Cybervictimization is defined as an intentional abusive behavior repeatedly received by a victim who has difficulty defending him/herself [10]. It is performed by more powerful peers engaging in hostile, hurtful, and damaging behaviors using digital technologies [11]. As with traditional bullying, one of the characteristics of cyberbullying is the power imbalance between the bully and the victim [12], which is altered and more pronounced than in bullying. Anonymity, the constant possibility of threat, and a potentially larger audience are features of online technology that contribute to this power imbalance. These factors influence power imbalance, and, therefore, these technologies give power to bullies and take power away from victims [13,14]. Cyberbullying includes different types of behaviors, from relational to picture-based attacks. The behavior can be more or less direct: direct cyberbullying, where the perpetrator interacts directly and immediately with the victim using direct chat—for example, sending insulting messages or pictures—and indirect cyberbullying, where the perpetrator communicates through public or semi-public channels, posting negative comments or images about the victim in online social networks [15].

Online sexual harassment encompasses a wide range of behaviors that use digital content (images, videos, posts, messages, pages) on a variety of different platforms (private or public). It can make a person feel threatened, exploited, coerced, humiliated, upset, sexualized, or discriminated against [16]. Regarding online sexual harassment, it is possible to identify some core characteristics of this phenomenon: online sexual harassment has an abusive connotation, as it is perceived as unwanted by the victim, and it can occur in three main typologies (verbal, visual, and cybersex), and even a single episode is enough to experience victimization [17]. In terms of relational behaviors, online sexual harassment includes unwanted sexual solicitations and non-consensual sharing [17]. Online sexual harassment among peers is a form of peer aggression frequently experienced by adolescents [18,19,20], but prevalence data are inconsistent and contradictory due to the many differences in measurement and sampling designs, while data on cyberbullying are much more transparent.

Bullying and cyberbullying are behaviors that arise intending to find the “weak point” of the victim: ethnic, prejudicial, disability-based, or sexual bullying. On the other hand, online sexual harassment already has a sexual connotation, and its fundamental characteristic is how the act is received (i.e., whether it is unwanted or unwelcome). The sexual connotation of the behavior could be due to misconceptions about masculinity and a discriminatory and objectified view of women, resulting in the adolescent not yet knowing how to relate to others in the context of romantic relationships and/or not knowing how to self-regulate impulses.

The two behaviors seem to be highly correlated, both face to face [20] and online, although in this last context, little is known [21]. A consistent co-occurrence has been reported by few studies: one study reported whether 9% of middle and high school students were involved in all the traditional and cyber forms of bullying and sexual harassment [22], and, coherently, 7% of Swedish adolescents were victimized by all four forms of victimization (traditional and online bullying and sexual harassment) [23]. Cyberbullying and cyber sexual harassment (such as traditional bullying and face-to-face sexual harassment) may be variations of the same in-person behaviors, explaining their covariant perpetration as they are different forms of aggressive and dominant behaviors. Given that the sexual connotation of aggressive behaviors becomes more important in the group dynamics as adolescents become more sexually mature [20], their co-occurrence during this period is particularly evident. It is often difficult to make a clear distinction between the two forms of victimization. Sexual harassment can include in its definition behaviors that constitute sexual harms (e.g., sending unwanted sexual content or asking someone to do something sexual) [24]. Cyberbullying is often sexualized (i.e., bullying someone because of their physical appearance or sexual orientation, spreading false rumors about girls’ sexual reputations), and it is often investigated through self-report measures where most adolescents consider both non-sexualized and sexualized forms [22,25], which could be a reason for the overlap between the two phenomena.

Another reason for their co-occurrence could be related to the possibility that online sexual harassment and cyberbullying may share a common set of risk factors.

### Risk and Protective Factors: An Ecological Approach 

Online sexual harassment among peers (OSH-P) and cyberbullying (CB) share many common risk factors at different levels of the social-ecological model (i.e., individual, relationship, school, and community) [26]. At the individual level, they include high impulsivity, low empathy, low self-esteem, anger, and traditional beliefs about masculinity [27,28,29,30]. Victims of OSH-P and CB have both reported greater prior problem behaviors, including marijuana use, alcohol use, and depressive symptoms [24]. In terms of relational factors, we find gender, family structure, family conflict and hostility, low parenting and poor monitoring, low social support, and delinquent peer associations [30,31,32,33,34]. Conversely, exposure to neighborhood violence and lower school belonging, or a sense of peer group belonging, are common factors at the community level [31,33,35]. Indeed, in the school context, climate, sense of belonging, and relationships with peers are extremely important factors. The development of positive peer relationships contributes to maintaining a healthy school climate, and many studies have shown how this can positively influence student outcomes [36,37,38]. Research has also found important links between positive peer relationships, peer acceptance and interaction, and lower victimization rates [36,39]. Peer social connectedness is an important predictor of developing prosocial behaviors and managing conflict, which reduces the likelihood of engaging in aggressive behaviors, either perpetrating or victimizing [38].

Although several studies have addressed risk factors for the two phenomena separately by trying to compare the different pictures [24,40], to date, no studies have been published about whether a common set of risk factors might predict longitudinally cybervictimization and online sexual harassment, controlling for their co-occurrence.

Considering that cyberbullying and OSH-P are both forms of online peer victimization, it is important to consider the extent to which these experiences are related and to understand their commonalities more accurately. This short longitudinal study aims to (a) provide data on the prevalence of OSH-P and cybervictimization within a sample of Italian adolescents, evaluating their co-occurrence, and (b) identify individual (i.e., emotional and problem behaviors) and contextual risk factors (school climate, social support) for OSH-P and cybervictimization, controlling for their co-occurrence using a path analysis model.

## 2. Materials and Methods

### 2.1. Participants and Measures

The sample included a total of 697 adolescents (42.3% females) enrolled in Grade 9 of high schools in Tuscany (Italy), who participated in at least one of two time points of data collection. The mean age was 15.17 years (SD = 0.68) at the baseline, ranging from 14 to 19 years. Most students were born in Italy (81.7%), and the remaining adolescents (18.3%) came from different countries.

This research is based on the fourth and fifth waves of a longitudinal project of national interest (PRIN). An initial number of 60 schools were contacted by e-mail, of which 21 decided to participate in the project. Data collection was held during regular school class hours between January and March 2022 (Wave 4) and May and June 2022 (Wave 5) through an online questionnaire. Qualified researchers followed the administration of the questionnaire in the classrooms by connecting remotely. The consent of students who had already reached 14 years of age was obtained in advance. Participation was voluntary, and the questionnaire was anonymous. Students did not receive awards or incentives for participation. In this study, only the control sample was considered.

The study received the approval of the Research Ethics Commission of the University of Florence and was conducted according to the Ethical Principles of Psychologists and Code of Conduct.

### 2.2. Measures

The peer sexual cybervictimization (SCV)—revised [41] was used to assess online sexual harassment among peers. This scale, composed of 12 items, measures 3 subdimensions of OSH-P: ambiguous sexual cybervictimization, personal sexual cybervictimization, and non-consensual sharing of sexual content. For each item proposed, the participants had to indicate how often they had been the victim of that behavior using a scale from 1 (Never) to 5 (Daily). The reliability of the overall scale, computed from the original items, was good when ω = 0.85 (wave 4), ω = 0.88 (wave 5). Considering the non-normal distribution of the data, responses were recoded as categorical (0 = absence of victimization, 1 = at least once as a victim).

Cybervictimization was measured with the Florence Cyberbullying and Cybervictimization Scales—Short Version Revised [42]. The scale consists of four items asking how often respondents have experienced online aggressive behavior as a victim (e.g., “I have received threats and insults on the internet (social networks, chats, blogs, etc.)”; “I have received/seen embarrassing or intimate photos or videos of me (on social networks, chats, blogs, etc.)”) during the past couple of months. Each item was rated on a 5-point scale ranging from 1 (never) to 5 (several times a week). Internal reliability, computed from the original items, was acceptable when ω = 0.64 (wave 4) and ω = 0.68 (wave 5). Considering the non-normal distribution of the data, responses were recoded as categorical (0 = absence of victimization, 1 = at least once as a victim).

The Strengths and Difficulties Questionnaire (SDQ) [43] was used to assess emotional and behavioral problems. The scale consisted of a brief self-report questionnaire of 25 items, analyzing conduct problems such as hyperactivity, emotional symptoms, peer problems, and prosocial behavior. Every subscale was assessed by 5 items rated on a 3-point Likert scale (0 = not true, 1 = somewhat true, or 2 = certainly true). The internal reliability (McDonald’s Omega) of subscales ranged between 0.55 and 0.70.

The Georgia Health School Survey—(GHSS) [44] was used to assess school climate. This scale analyzes sub-dimensions of school climate such as school connectedness, peer social support, adult social support, and social and civic learning. The scale consists of 19 items; for each item, students have to indicate how much they agree with every sentence on a 4-point Likert scale (0 = completely disagree, 4 = completely agree). The internal reliability (McDonald’s Omega) of the subscale ranged between 0.77 and 0.89.

### 2.3. Data Analysis

First, descriptive statistics and correlation estimates were computed using SPSS software. Second, using Mplus 7.0 software, a path analysis model was used to test the association between psychological problems, school climate, cybervictimization, and OSH-P. Given that the model was saturated, fit indices were not reported. Correlations between all the variables under study were estimated, but they were not reported in the model except for those between the two outcomes (i.e., cybervictimization and online sexual harassment among peers at Wave 5). The two outcome variables, CV and OSH-P, were recorded as categorical (0 = absence of victimization, 1 = at least once a victim). This decision depends both on reasons related to the non-normal distribution of the variable and the fact that a single episode of CV is sufficient to generate many repetitions of victimization, as there are no temporal or geographical limits [45], and that one of the characteristics of online sexual harassment is that it can happen only once. Consequently, the variables were declared as categorical in Mplus, and the estimator weighted least square mean and variance (WLSMV) was used.

## 3. Results

### 3.1. Data Prevalence

Descriptive statistics and correlation estimates are reported in Table 1. Regarding data prevalence, in the first survey (Wave 4), 41.3% of respondents reported having been the victim of OSH-P at least once. The data for cybervictimization were very similar (42.1%). Being a victim of cybervictimization was positively associated with being a victim of OSH-P (*ρ*s = 0.426**). In the second survey (Wave 5), the frequency of both OSH-P and victimization decreased while remaining very similar for the two behaviors: OSH-P (37.7%) and cybervictimization (36.6%). Again, being a victim of cybervictimization was positively associated with being a victim of OSH-P, although to a lesser extent (*ρ*s = 0.371**). Concurrent analysis showed that 27.7% (Wave 4) and 22.4% (Wave 5) of the participants were involved in both online sexual harassment and cybervictimization. Specifically, 67.2% (Wave 4) and 59.5% (Wave 5) of participants who were cybervictimized were also victims of online sexual harassment.

### 3.2. Path Analysis Model

**Figure 1:**
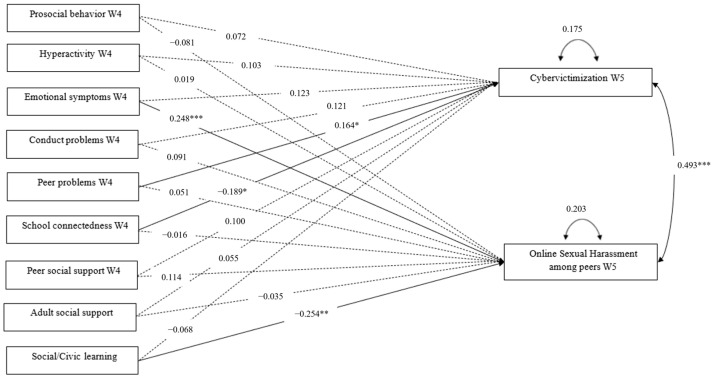
Tested model of the study. * *p* < 0.05; ** *p* < 0.01; *** *p* < 0.001.

Regarding the path analysis, as shown in Table 2 and Figure 1, the results show that online sexual harassment and cybervictimization are positively correlated with each other (β = 0.493***, SE = 0.064) and seem to be explained by different factors. Specifically, cybervictimization at Wave 5 is predicted by the presence of problematic peer relationships (β = 0.164*, SE = 0.068) and by the lack of school connectedness (β = −0.189*, SE = 0.086) at Wave 4. The presence of OSH-P in Wave 5 is instead predicted by the presence of emotional symptoms (β = 0.248***, SE = 0.077) and the absence of social and civic norms (β = −0.254**, SE = 0.085) in Wave 4.

## 4. Discussion

This study aims to first provide data on the prevalence of OSH-P and cybervictimization and their co-occurrence within a sample of Italian adolescents and then to identify individual and contextual risk factors for OSH-P and cybervictimization.

In line with our hypothesis, the results show that 41.3% of respondents reported having been the victim of OSH-P at least once in Wave 4 and 37.7% in Wave 5. The data for cybervictimization were very similar, with a prevalence of 42.1% in Wave 4 and 36.6% in Wave 5. The involvement in victimization (both for cybervictimization and for OSH-P) decreased over time, and this could be as, at the end of the year, the students, mostly attending the first year of secondary school, have gained greater mutual understanding and developed better group cohesion. Being a victim of cybervictimization was positively associated with being a victim of OSH-P. Overall, the co-occurrence ranged between 27.7% (Wave 4) and 22.4% (Wave 5) in the general population, whereas higher percentages of overlap were found for the students involved: 67.2% (Wave 4) and 59.5% (Wave 5) of participants who were cybervictimized were also victims of online sexual harassment.

These findings are consistent with previous studies that have reported a consistent co-occurrence between the two phenomena [22,23]. Cybervictimization and online sexual harassment represent variations of the same in-person behavior and are merely extensions of offline or face-to-face sexual harassment and bullying [23]. OSH-P and cybervictimization are behaviors that involve an aggressor and a victim in an online context. The anonymity, asynchrony, and lack of geographic and temporal limits of the Internet create a strong power imbalance between victim and aggressor, making even a single episode sufficient to experience victimization [25,46,47,48,49]. Moreover, for both phenomena, victimization can occur through various forms of digital content (images, videos, posts, messages, sites) on a variety of different platforms (private or public). Furthermore, some behaviors are also shared between the two phenomena: sexual harassment includes sexual harms [24], while cyberbullying is often sexualized, and no distinction is made in the measurement between non-sexualized and sexualized forms [22,25]. This evidence supports that the poly-victimization could explain the overlap between OSH-P and CV.

The findings showed that OSH-P and cybervictimization are positively correlated with each other but seem to be explained by different factors. Specifically, cybervictimization is predicted by the presence of problematic peer relationships and by the lack of school connectedness. The presence of OSH-P is instead predicted by the presence of emotional symptoms and the absence of social and civic norms.

Although cybervictimization takes place online, it remains a class phenomenon, which is often the online extension of a pre-existing offline form of victimization [45,50,51]. For this reason, having problematic relationships with peers (i.e., being isolated, having no friends, not being liked by peers, etc.) is a strong risk factor for cybervictimization [52,53]. In fact, bullies often choose victims who are insecure and submissive, targeting socially marginalized classmates who are more likely to assert their power than their peers [54]. The lack of school connectedness emerges as a predictor, as factors related to group norms and the characteristics of the school and teachers in general can greatly influence the explanation of differences in the prevalence of the phenomenon.

On the other hand, OSH-P appears to be predicted by the presence of emotional symptoms (i.e., sadness, crying, fear, and worrying a lot). This finding is consistent with the literature [27,28,30]. In terms of the lack of social and civic norms, they become predictors of the phenomenon that manifests itself in a school environment where not all children are treated equally; discrimination is indeed an important risk factor for sexually connoted behaviors [55,56]. Women and the LGBTQ+ community are more likely to be victims of online sexual harassment, and online sexual harassment has a gender bias, so girls are more likely to be victims than boys [22].

### Limits and Future Directions

This study has two main strengths. First, it is one of the few longitudinal studies that has analyzed the effects of online sexual harassment separately from offline sexual harassment. Second, it is the first study in the Italian context to examine this form of victimization and to use a validated measure to detect the prevalence of the phenomenon.

Despite the strengths of this study, it is important to consider some limitations when interpreting the results. First, the study did not consider specific risk factors for online sexual harassment, such as exposure to pornography, rejection of sexual harassment, and early sexual initiation [30]. Second, the sample had a very limited age range, as most of the students who participated in the survey were in their first year of high school. Third, the model accounted for the general sample by not distinguishing between genders. Future studies could investigate whether the association between the included variables differs in the male versus female sample and whether gender might play a moderating role between the included risk factors and outcome variables (i.e., OSH-P and CV). Finally, only social and contextual factors were considered as risk factors in predicting the two phenomena. Future studies could consider other types of risk factors, such as risky online behavior. In fact, risky online behaviors, such as sexting and exhibitionism, as well as the indiscriminate expansion of one’s network, predict the increased risk of online sexual victimization among adolescents [57].

## 5. Conclusions

OSH-P and cybervictimization are related phenomena; being a victim of cybervictimization is positively associated with being a victim of OSH-P. However, risk factors are different: cybervictimization remains more linked to a classroom context, where the dimension of group and school dynamics and the relationship with peers are important predictors. OSH-P is less related to class factors and more related to social factors (non-discriminatory environment, etc.), although individual characteristics remain important, so this form of victimization is often associated with depressive and anxiety symptoms.

Considering age, adolescents are less aware of the risks they run by disseminating and publishing intimate material on the Internet; many cross-sectional studies with children and young people of different ages indicate that concerns about online privacy increase with age [58,59,60,61]. In fact, it is not surprising that there is a correlation between being a victim of online sexual harassment between peers and being a victim of cyberbullying, especially in a period of sexual and social development. At this stage, sharing and publishing intimate material on the Internet could be a (risky) way of experimenting with one’s identity. Furthermore, this is the age in which the first romantic relationships are usually established, so the “aggressor” could also use this form of communication as an attempt to approach a potential partner, as he is still immature. However, it is important to remember how sexual harassment behaviors are strongly influenced by stereotypes related to inequality between women and men, and this usually produces more negative outcomes and experiences for women and girls [16]. The sexual connotation of harassment does not refer only to sexual acts but also to gender harassment. Belonging to a specific gender rather than to another can be a cause of harassment. In this case, we can notice a gender bias: a man (or a boy) who displays high levels of sexual behavior increases his social status among peers, but a woman (or a girl) can more easily be negatively attacked (i.e., slut-shaming) or receive negative judgments for the same behaviors [62]. Girls are also more concerned with their physical appearance and therefore tend to use social networks and the online environment in general to strengthen their self-esteem. Behaviors such as sexy online self-presentation and concerns about body image make girls more at risk of online sexual harassment [63,64,65].

Future studies should replicate these findings by expanding the sample to a larger population, including the role of gender, and considering risk factors more closely related to online sexual harassment.

Finally, the findings suggested that, given their co-occurrence, cybervictimization and online sexual harassment should be prevented using cross-cutting strategies on general aggression and victimization. However, more attention to social and contextual components within the interventions is necessary for cybervictimization.

## Figures and Tables

**Table 1:** Correlations between all study variables.

Variables	1.	2.	3.	4.	5.	6.	7.	8.	9.	10.	11.
1. Pros. behav. W4	1										
2. Hyperact. W4	−0.209 ***	1									
3. Emot. sympt. W4	0.166 ***	0.417 **	1								
4. Conduct probl. W4	−0.282 ***	0.458 **	0.361 **	1							
5. Peer probl. W4	−0.154 ***	0.123 **	0.349 **	0.187 **	1						
6. School connect. W4	0.315 ***	−0.253 **	−0.170 **	−0.235 **	−0.314 **	1					
7. Peer soc. supp. W4	0.359 ***	−0.107 **	0.011	−0.110 **	−0.406 **	0.641 **	1				
8. Adult soc. supp. W4	0.271 ***	−0.181 **	−0.145 **	−0.295 **	−0.227 **	0.553 **	0.507 **	1			
9. Soc./Civ. learn. W4	0.293 ***	−0.160 **	−0.087 *	−0.233 **	−0.285 **	0.613 **	0.659 **	0.631 **	1		
10. CV W5	−0.013	0.202 **	0.246 **	0.198 **	0.205 **	−0.185 **	−0.076	−0.099 *	−0.125 **	1	
11. OSH-P W5	−0.096 *	0.180 **	0.258 **	0.219 **	0.165 **	−0.178 **	−0.108 *	−0.192 **	−0.233 **	0.371 **	1
Mean	1.380	0.828	0.757	0.514	0.461	2.757	3.242	3.017	3.033	41.3%	42.1%
Sd	0.457	0.449	0.548	0.376	0.390	0.624	0.670	0.761	0.686	-	-

*Note:* Pros. behav. = Prosocial behavior; Hyperact. = Hyperactivity; Emot. Sympt. = Emotional symptoms; Conduct probl. = Conduct problems; Peer probl. = Peer problems; School connect. = School Connectedness; Peer soc. supp. = Peer social support; Adult soc. supp. = Adult social support; Soc./Civ. learn. = Social/Civic learning; CV = Cybervictimization; OSH-P = Online Sexual Harassment among Peers. CV and OSH-P at W5 were dichotomized; thus, we report the proportion. * *p* < 0.05; ** *p* < 0.01; *** *p* < 0.001.

**Table 2:** Estimates from the path model predicting cybervictimization and OSH-P at Wave 5 using individual and school risk factors.

Outcome	Predictors	β	SE	95%C.I.	*p*-Value
CV W5	Pros. behav. W4	0.072	0.073	−0.048, 0.192	0.327
	Hyperact. W4	0.103	0.068	−0.009, 0.215	0.130
	Emot. Sympt. W4	0.123	0.079	−0.007, 0.252	0.119
	Conduct probl. W4	0.121	0.067	0.012, 0.231	0.069
	Peer probl. W4	0.164	0.068	0.052, 0.276	0.016
	School connect. W4	−0.189	0.086	−0.330, −0.048	0.028
	Peer soc. supp. W4	0.100	0.089	−0.063, 0.264	0.312
	Adult soc. supp. W4	0.055	0.086	−0.086, 0.196	0.519
	Soc./Civ. learn. W4	−0.068	0.094	−0.222, 0.087	0.473
OSH-P W5	Pros. behav. W4	−0.081	0.071	−0.198, 0.036	0.256
	Hyperact. W4	0.019	0.067	−0.092, 0.129	0.781
	Emot. Sympt. W4	0.248	0.077	0.122, 0.374	0.001
	Conduct probl. W4	0.091	0.068	−0.021, 0.203	0.183
	Peer probl. W4	0.051	0.069	−0.062, 0.165	0.457
	School connect. W4	−0.016	0.085	−0.157, 0.124	0.848
	Peer soc. supp. W4	0.114	0.095	−0.043, 0.271	0.231
	Adult soc. supp. W4	−0.035	0.080	−0.166, 0.096	0.660
	Soc./Civ. learn. W4	−0.254	0.085	−0.394, −0.115	0.003

Pros. behav. = Prosocial behavior; Hyperact. = Hyperactivity; Emot. sympt = Emotional symptoms; Conduct probl. = Conduct problems; Peer probl. = Peer problems; School connect. = School Connectedness; Peer soc. supp. = Peer social support; Adult soc. supp. = Adult social support; Soc./Civ. learn. = Social/Civic learning; CV = Cybervictimization; OSH-P = Online Sexual Harassment among Peers. CV and OSH-P at W5 were dichotomized; thus, we report the proportion.

## Data Availability

The data supporting this study’s findings are available on request from the corresponding author. The data are not publicly available due to privacy or ethical restrictions.
